# Weight bias: a call to action

**DOI:** 10.1186/s40337-016-0112-4

**Published:** 2016-11-07

**Authors:** Angela S. Alberga, Shelly Russell-Mayhew, Kristin M. von Ranson, Lindsay McLaren

**Affiliations:** 1 Werklund School of Education, University of Calgary, 2500 University Dr NW, T2N 1N4 Calgary, AB Canada; 2 Department of Psychology, University of Calgary, 2500 University Drive NW, T2N 1N4 Calgary, AB Canada; 3 Community Health Sciences, Cumming School of Medicine, University of Calgary, TRW Building, 3rd Floor, 3280 Hospital Drive NW, T2N 4Z6 Calgary, AB Canada

**Keywords:** Weight stigma, Weight prejudice, Overweight, Eating behaviours

## Abstract

Weight-related issues (including excess weight, disordered eating and body concerns) are often considered as comprising distinct domains of ‘obesity’ and ‘eating disorders’. In this commentary we argue that the concept of weight bias is an important variable when considering wellbeing across the spectrum of weight-related issues. We make the following six points in support of this argument: i) weight bias is common and has adverse health consequences, ii) shaming individuals for their body weight does not motivate positive behaviour change, iii) internalized weight bias is particularly problematic, iv) public health interventions, if not carefully thought out, can perpetuate weight bias, v) weight bias is a manifestation of social inequity, and vi) action on weight bias requires an upstream, population-level approach. To achieve sustainable reductions in weight bias at a population level, substantive modifications and collaborative efforts in multiple settings must be initiated. We provide several examples of population-level interventions to reduce weight bias.

## Background

*“‘My therapist tells me not to talk about my weight and that my body is fine. But my doctor keeps weighing me and says that I need to lose weight,’ Ms. Schaefer said” (New York Times, October 10, 2013).*

This quote illustrates a traditional disjoint in perspectives between practitioners in the fields of obesity and eating disorders [1], with different research priorities and approaches to prevention and management. However, a growing body of scholarship acknowledges that these weight-related domains are in fact related: obesity and eating disorders co-occur in individuals [2] and have risk factors in common [3]. It has been suggested that an integrative approach to weight-related issues, which merges knowledge from the fields of obesity and eating disorders, is central to effective prevention [4–6]. In this commentary we argue that the concept of weight bias is an important variable to consider in an integrative approach to wellbeing across the spectrum of weight-related issues. Although there are significant literatures devoted to obesity and eating disorders, reviewing those literatures is beyond the scope of this paper. Rather, our focus is to address weight bias and offer potential population-level interventions to reduce it.

*Weight bias* is defined as “negative weight-related attitudes, beliefs, assumptions and judgments toward individuals who are overweight and obese” [7]. We extend this definition to include individuals of low as well as high weights. *Weight-related issues* include obesity and eating disorders, but importantly also include disordered eating and non- or sub-clinical variants or symptoms, such as overweight, body image dissatisfaction, restrained eating, disinhibited eating, emotional eating, and compensatory behaviors. The causes of weight-related issues are complex and multi-factorial [8]. For the purpose of this paper, we emphasize the important role played by social, economic, and political influences [9, 10]. Though individual agency plays a role, fixation on individuals’ responsibility for weight serves to oversimplify and overstate [11].

There are at least six reasons that weight bias provides a useful and potentially powerful focal point for an integrated approach to wellbeing across the spectrum of weight-related issues.

### (1) Weight bias is common and has adverse health consequences

Weight bias impacts people across the weight spectrum [12, 13] and has increased over time [14]. People classified as ‘overweight’ or ‘obese’ have been shamed for their high weight [15, 16]. There is also some evidence that adolescents categorized as ‘underweight’ [16] and individuals with eating disorders (e.g., anorexia, bulimia nervosa and binge eating disorder) have experienced weight bias [13]. Weight bias reflects, in part, that unlike other conditions, body weight is a physical characteristic that is visible. Weight bias has been associated with adverse health outcomes including anxiety, stress, depression, low self-esteem and body image issues [15, 17, 18]. Though disease consequences and mortality [19–21] of very high levels of excess weight have been documented, it has been proposed that the *stigma* associated with weight may actually be causing some of the negative health outcomes associated with excess weight rather than the excess weight itself [22], including increased mortality risk [23]. There is a need to better balance these two sets of consequences when addressing weight-related issues.

### (2) Shaming individuals for their body weight does not motivate positive behaviour change

Evidence indicates that shaming individuals with weight-related issues does not motivate positive behaviour changes. To the contrary, experiencing weight bias could lead to the development of eating disorders and/or obesity. For example, individuals with obesity experiencing weight stigma often turn to unhealthy eating behaviors in line with eating disorder symptomatology, such as fasting, extreme dieting, frequent episodes of binge eating, and compulsive exercise [24–26]. Experiencing weight bias can also promote the avoidance of exercise (e.g., avoiding exercising in public for fear of being shamed for their weight) [27, 28] and maladaptive eating habits (e.g., binge eating related to the emotional stress of experiencing bias) [29, 30] that could promote weight gain.

### (3) Internalized weight bias is particularly problematic

Internalized weight bias, defined as individuals’ belief that they deserve the stigma and discriminatory treatment they experience as a result of having overweight or obesity [31], is particularly worrisome. People with eating disorders typically report high levels of internalized weight bias wherein they have an intense fear of being fat and a fear that being fat would negatively affect their life [32]. People with obesity also experience internalized weight bias [17]. These observations illustrate how weight bias is implicated across the spectrum of weight-related issues, but may play different roles and manifest in different ways. Internalized weight bias is strongly associated with psychological maladjustment and eating pathology, including depression, poor body image [20], low self-esteem, avoidance of preventive health care [15] and lack of engagement in primary health care settings [33].

### (4) Public health interventions, if not carefully thought out, can perpetuate weight bias

A weight-centric approach, in which weight is viewed as a proxy for health and beauty, has contributed to individuals with overweight or obesity experiencing weight bias and discrimination with increasing frequency and intensity [26]. Though adverse health correlates of obesity such as morbidity [34] and mortality [23] have been documented, the health implications associated with lower levels of “excess weight” are not clear and may be overstated [19]. It is not clear whether or the extent to which the adverse psychological and physical consequences of obesity are related to excess weight itself and/or weight bias. There is evidence to suggest that negative psychological outcomes are linked to experiencing weight bias even after controlling for age, gender, obesity onset and body mass index [35, 36]. The focus on the health consequences of obesity has led to public ‘fat panic’ [25] through media portrayals and public health policies, programs, and campaigns [37] that glamorize thinness and demonize fatness. For example, the Children’s Healthcare of Atlanta, U.S.A. launched Strong4Life, described as “a wellness movement designed to ignite societal change and reverse the epidemic of childhood obesity and its associated diseases in Georgia”. This controversial childhood obesity public “health campaign” used images of children with obesity on billboards and websites with captions such as *“Warning: Big bones didn’t make me this way, big meals did”* and *“Warning: chubby kids may not outlive their parents”*. Such initiatives have been criticized for their weight-stigmatizing messages [12]. Puhl et al. also surveyed a nationally representative sample of US adults to determine how obesity-related public health media campaigns are ranked. Participants responded least favorably to the messages that were publicly criticized for their stigmatizing content and showed fewer intentions to comply with the message content [38]. On the contrary, this study showed that participants were more motivated by messages that made no mention of the word ‘obesity’ but focused on healthy behaviors without relating them to body weight.

Efforts that promote weight loss to ‘improve’ one’s appearance can perpetuate weight bias if their messages equate thinness with health and/or beauty. Promoting weight loss to achieve these standards could promote unhealthy preoccupations with weight and size and the development of disordered eating patterns. It has been shown that media images of thin bodies play an important role in the etiology of eating disorders [39].

### (5) Weight bias is a manifestation of social inequity

Social inequity refers to unequal access, opportunities, rewards, and therefore outcomes for different social groups that are unfair and unjust [40, 41]. Socially-defined groups of people, such as people defined by their high or low body weight, can be treated unequally. Weight bias is a manifestation of social inequity because people belonging to the ‘large bodies’ social group are not treated equally to the ‘small bodies’ social group in various sectors in society (e.g., employment, education, healthcare) [15, 42]. People belonging to the ‘small bodies’ social group have more privileges and are rewarded differently than people belonging to the ‘large bodies’ social group [15, 42]. Stigma has been identified as a fundamental cause of population health inequities [43]. Weight bias contributes to harm and violation of human rights [44], in that prevalent stereotypes are often unchallenged and people living with obesity are vulnerable to unfair treatment simply because they have large bodies [15]. Numerous studies show that children and adults living with obesity are treated unequally because of their size at school, at work, in interpersonal relationships and within the healthcare system [15]. It has been argued that weight bias is a socially acceptable form of prejudice today [45]. To illustrate, whereas “race, national or ethnic origin, colour, religion, sex, age or mental or physical disability” are protected in the Canadian Charter of Rights and Freedoms [46], weight is not. Similarly, there are no federal laws prohibiting weight bias in other countries including the United States [47], despite public support for laws prohibiting weight discrimination [22, 48].

Although more research is needed on the root causes of weight bias [49], one of the causal explanations for having weight-biased attitudes is holding the belief that obesity is ‘controllable’ by individuals [50]. Although individual choice and agency are recognized in weight management, a society that highly values individualism may greatly overstate the ‘controllability’ of obesity which sets the stage for weight bias. To some extent, body weight has a biological underpinning whereby most individuals who lose weight cannot sustain weight loss over the long term [51, 52]. Furthermore, several national organizations have recognized obesity as a chronic disease, including the American Medical Association [53] and the Canadian Medical Association [54], supported by research evidence showing the genetic basis and the complexity of obesity lies beyond the individual [8, 55]. As noted above, evidence of the prevalence and consequences of weight bias suggest that treating people disrespectfully because of their weight is harmful to their physical and mental health and it does not result in positive behavior change related to weight loss [56, 57]. Therefore, weight bias does not appear to be justified as a public health tactic to address obesity [22]. All people, regardless of body size, deserve respect, equity, and dignity, and to live without stigma and discrimination.

### (6) Action on weight bias requires an upstream, population-level approach

Since weight bias can be experienced across the weight spectrum, and experiencing weight bias can lead to the development and persistence of weight and body-related concerns across the population, a population-level approach is necessary to ensure respect for people of all shapes and sizes. The traditional approach to reducing stigma associated with obesity has been to raise awareness and educate individuals primarily in clinical contexts [58] by improving knowledge on the multifactorial etiology of obesity, increasing awareness of weight bias and its negative implications, and providing sensitivity training on the prevention and management of obesity. It is unclear whether these initiatives promote positive behaviour changes or worsen attitudes about weight over the long term. Evidence is limited about whether these sorts of changes are sustainable and whether they impact populations by addressing deeper, fundamental causes of weight bias. Adopting a population health approach allows us to build on the existing prevention literature that is mostly focused on downstream approaches to prevention [59, 60].

The Nuffield Council on Bioethics’ intervention ladder [61] (Table 1) illustrates the breadth of intervention activities that one could consider within a population/public health approach. In that framework, educational initiatives providing information are identified as the second lowest level, which corresponds to more limited capacity to positively impact population health and health inequities. We argue that upstream population-level approaches to addressing weight bias, such as activities higher up on the intervention ladder, should be considered, with the potential benefits being balanced against the increasing level of intrusion or coercion. Prevention efforts should not be giving conflicting messages to confuse the population but should focus on promoting health and wellbeing. Examples of higher-level intervention ideas aligned with an upstream population health approach to wellbeing across the spectrum of weight-related issues are provided in Table 1. To complement suggested activities in Table 1, it is important for people working in the fields of obesity and eating disorders to reflect on their own assumptions.

**Table 1:** Examples of interventions to prevent weight bias, organized using the Nuffield Council on Bioethics’ intervention ladder as a framework [52]

Government action	Examples in the field of weight bias (Research locations)
Restrict choice.	• Develop legislation to prohibit weight discrimination [48] (U.S.A., Canada, Australia, Iceland)
	• Implement anti-discrimination laws against bullying in schools and weight discrimination in the employment and healthcare sectors [62] (U.S.A.)
	• Mandatory post-secondary curricula and appropriate training on weight-related issues for pre-service student teachers, health professionals and public health practitioners [58, 62, 63] (Canada, U.S.A., Iceland, Australia)
	• Formal training for coaches to prevent eating disorders in sports [62] (U.S.A.)
	• Mandatory implementation of evidence-based body appreciation, media literacy and eating disorder prevention programs in schools [62, 64] (U.S.A.)
	• Ban digital modification of images that glamorize thinness in women and muscularity in men in the media [65] (Australia)
Guide choice through disincentives.	• Implement penalties for evidence of weight discrimination in employment, healthcare and education sectors (e.g., charging schemes in the employment and healthcare sectors, exclusion from extra-curricular activities for youth in schools)
Guide choice through incentives.	• Offer awards, fiscal or other incentives for the promotion of wellbeing and body inclusivity in the education, healthcare and employment sectors (e.g., a school board could offer an award or recognition for schools that implement body inclusivity in their teaching and learning practices)
Guide choice through changing the default policy.	• Devise media and journalism guidelines for prohibiting gender-based and weight-based stereotypes in the media [66] (U.S.A.) (e.g., stop portraying women of size eating ice cream to cope with mental health issues)
	• Depict positive stereotypes of people living with obesity in the media [67] (U.S.A.)
Enable choice.	• Modify the built environment to accommodate individuals of all weights [68] (U.S.A.) (e.g., chairs in waiting rooms, staircases, airplane seats, hospital beds, clothing uniforms and exercise equipment)
	• Offer an evidence-based school program geared towards positive body image, acceptance of body diversity and prevention of weight-related issues [64] (U.S.A.)
Provide information.	• Create flyers and posters that promote positive body image and body diversity and distribute them in schools
	• Disseminate population health campaigns to address weight bias [69] (Australia)
Do nothing or simply monitor the situation	• Monitor the prevalence of weight bias in different sectors (i.e., education, healthcare, employment)
	• Do nothing

Note: Strategies for evaluation of the effectiveness and cost of these initiatives must also be incorporated

## Conclusion

Traditionally, weight-related issues such as obesity and eating disorders have been treated as separate and distinct research and practice domains. This commentary argues that the concept of weight bias is an important variable when considering wellbeing across the spectrum of weight-related issues. Sustainable reductions in weight bias at a population level necessitate substantive upstream modifications and collaborative efforts in multiple settings.
